# Racism is a motivator and a barrier for people of color aspiring to become midwives in the United States

**DOI:** 10.1111/1475-6773.14037

**Published:** 2022-07-23

**Authors:** Renee Mehra, Amy Alspaugh, Jennie Joseph, Bethany Golden, Nikki Lanshaw, Monica R. McLemore, Linda S. Franck

**Affiliations:** ^1^ Department of Family Health Care Nursing School of Nursing, University of California San Francisco California USA; ^2^ College of Nursing University of Tennessee Knoxville Tennessee USA; ^3^ Commonsense Childbirth Inc. Orlando Florida USA

**Keywords:** birth equity, concordant care, maternal health, midwifery, racism

## Abstract

**Objective:**

To understand motivators and barriers of aspiring midwives of color.

**Data Sources and Study Setting:**

Primary data were collected via a national online survey among people of color in the United States interested in pursuing midwifery education and careers between February 22 and May 2, 2021.

**Study Design:**

Cross‐sectional survey consisted of 76 questions (75 closed‐ended and 1 open‐ended questions) including personal, familial, community, and societal motivators and barriers to pursuing midwifery.

**Data Collection/Extraction Methods:**

We recruited respondents 18 years and older who identified as persons of color by posting the survey link on midwifery, childbirth, and reproductive justice listservs, social media platforms, and through emails to relevant midwifery and doula networks. We conducted descriptive and bivariate analyses by demographic characteristics and used exemplar quotes from the open‐ended question to illustrate findings from the descriptive data.

**Principal Findings:**

The strongest motivating factors for the 799 respondents were providing racially concordant care for community members (87.7 percent), reducing racial disparities in health (67.2 percent), and personal experiences related to midwifery care (55.4 percent) and health care more broadly (54.6 percent). Main barriers to entering midwifery were direct (58.2 percent) and related (27.5 to 52.8 percent) costs of midwifery education, and lack of racial concordance in midwifery education and the midwifery profession (31.5 percent) that may contribute to racially motivated exclusion of people of color. Financial and educational barriers were strongest among those with lower levels of income or education.

**Conclusions:**

Structural and interpersonal racisms are both motivators and barriers for aspiring midwives of color. Expanding and diversifying the perinatal workforce by addressing the financial and educational barriers of aspiring midwives of color, such as providing funding and culturally‐competent midwifery education, creating a robust pipeline, and opening more midwifery schools, is a matter of urgency to address the maternal health crisis.

**What is known in this topic**

- The United States is experiencing a maternal health crisis with maternal morbidity and mortality disproportionately affecting birthing people of color.
- Midwifery care and racially and culturally concordant care are important for improving health outcomes.
- Midwives of color are underrepresented in the United States and little is known about the motivators and barriers of aspiring midwives of color.

**What this study adds**

- In a large national sample, structural and interpersonal racism were found to be both motivators and barriers to entry into midwifery education and the midwifery profession by people of color.
- Providing racially concordant care for community members, reducing racial disparities in health, and personal experiences related to midwifery care and health care are important motivating factors.
- Main barriers to entering midwifery are direct and related costs of midwifery education and lack of racial concordance in midwifery education and the midwifery profession that may contribute to racially motivated exclusion.

## 1 INTRODUCTION

The United States is experiencing a maternal health crisis. Maternal mortality has been rising steadily since the 1990s, placing birthing people in the United States at a higher risk of death than in all other high‐income countries.^1^, ^2^ Severe maternal morbidities have increased by over 200 percent in the United States over the last several decades.^3^ Maternal illness and death are not equitably experienced by birthing people. Birthing people of color are disproportionately impacted, with Black and Native American or Alaska Native birthing people at least 2.5 times more likely to die during pregnancy, birth, and postpartum, and Hispanic, Native American or Alaska Native, and Black birthing people 1.2 to 1.7 times more likely to experience severe maternal morbidity than White birthing people.^4^, ^5^

Racial inequities in health are created and maintained by racism at multiple levels: structural, institutional, interpersonal, and intrapersonal.^6^ Structural racism includes laws and policies that contribute to differential access to power, privilege, opportunity, and resources; institutional racism includes policies and procedures in institutional contexts that contribute to differential access to power, privilege, opportunity, and resources; interpersonal racism includes discriminatory interactions between individuals; and intrapersonal racism includes internalized negative racialized ideas and attitudes.^6^

In the United States, inequitable outcomes in maternal and infant health are caused by a variety of factors rooted in a long history of racism, White supremacy, and reproductive coercion.^7^, ^8^, ^9^ Structural racism, manifest in racially discriminatory practices that exist in housing, employment, and educational opportunities, is associated with poorer birth outcomes.^10^, ^11^ Institutional racism contributes to racial inequities in maternal health through segregated access to high‐quality care.^12^ Interpersonal racism in the form of obstetric racism, is manifest in differential treatment (which may be intentional and coercive) that birthing people of color experience at the hands of health care providers and includes vital lapses in diagnosis, abusive and disrespectful treatment, and neglect, subjection to pain, and pressure to undergo procedures, leading to adverse negative health outcomes.^13^ Furthermore, explicit and implicit biases of health care providers play a large role in creating and sustaining such inequities within health care systems.^14^, ^15^ Intrapersonal racism and maternal and infant health is an understudied area of research, although associations between intrapersonal racism and obesity, chronic conditions, and stress in people of color suggest that this type of racism may also be detrimental to the health of birthing people of color.^16^, ^17^, ^18^

Midwifery care is an evidence‐based model of maternity and women's health care demonstrated to improve maternal health outcomes.^19^ In the United States, there are three professional designations of midwives (i.e., Certified Nurse‐Midwife, Certified Midwife, and Certified Professional Midwife), as well as traditional midwives.^20^, ^21^ National data are readily available only for Certified Nurse‐Midwives and Certified Midwives Certified Nurse‐Midwives comprise a small proportion of obstetric care providers, attending 9.8 percent of all births in 2019.^22^ The midwifery profession is predominately comprised of White midwives, who made up 85.52 percent of Certified Nurse‐Midwives/Certified Midwives in 2020, followed by Black (6.85 percent), Hispanic (4.73 percent), and American Indian or Alaska Native (0.58 percent) midwives.^23^ Midwifery students are similarly predominantly White, comprising 72.7 percent of students in 2018.^24^ Yet, of births attended by Certified Nurse‐Midwives in 2012, 56 percent were among White birthing people, 23 percent were among Hispanic birthing people, and 13 percent were among Black birthing people.^25^ Therefore, racial and ethnic identities of midwifery students and professionals in the United States do not reflect those of the population served.

Racially and culturally concordant care is increasingly recognized as an important component of holistic, patient‐centered care and for improving health outcomes.^26^, ^27^, ^28^ Racially concordant care is associated with greater health care utilization,^29^ improved patient‐physician communication,^30^ greater satisfaction with care,^31^ and reduced Black–White disparities in infant mortality.^32^ Therefore, increasing the number of midwives of color is an evidence‐based intervention to improve perinatal health in the United States. We use the term midwives of color as an expansive and inclusive term that includes those who self‐identify as Asian, Black, Indigenous, Latine, or other non‐White identities. To this end, it is important to understand why aspiring midwives of color are, or are not, entering the midwifery profession by identifying specific motivators and barriers to entry into the profession.

Our review of the literature revealed few studies of motivators and barriers for aspiring midwives of color in the United States. One study explored motivators for practicing midwives of color to provide care at a community birth center and identified three major findings: (1) importance of offering racially concordant care, (2) care motivated by racial justice, and (3) motivation to provide physical and emotionally safe care.^33^ These findings were similar to those from a study exploring what motivated people of color to become doulas,^34^ but diverged in many ways from results of research with White nurse‐midwives, who were motivated by an interest in maternity nursing and encouragement of other midwives.^35^, ^36^

Research on barriers to entering midwifery for people of color has not been directly explored, but some potential barriers can be inferred from research with midwives of color who expressed feelings of otherness and not belonging within the profession.^37^ Furthermore, the prevalence of interpersonal and institutional racism within midwifery education, professional organizations, and clinical settings may create barriers to the diversification of the midwifery workforce.^38^ Given the lack of research on motivators and barriers among people of color who aspire to become midwives, we conducted the “So, you want to be a midwife study?” A better understanding of motivators and barriers of aspiring midwives of color is essential to form policy recommendations to create and sustain a more diverse midwifery workforce and accelerate urgently needed improvements in maternal health care and outcomes in the United States.

## 2 METHODS

### 2.1 Research approach and study design

We conducted a survey among self‐identified people of color interested in pursuing all types of midwifery education and careers, between February 22 and May 2, 2021. Our study team included partners from Commonsense Childbirth Inc. and the University of California, San Francisco (UCSF). Commonsense Childbirth Inc. is a non‐profit organization located in Florida that operates maternity clinics and offers education and certification programs in perinatal health (https://commonsensechildbirth.org). The Commonsense Childbirth School of Midwifery is the first nationally accredited, Black‐owned, midwifery school in the United States. UCSF is a California public health sciences university (https://www.ucsf.edu). We collaborated to design the survey, analyze and interpret the data, and disseminate research findings.

### 2.2 Study population, setting, and recruitment

We invited individuals 18 years and older who identified as persons of color who resided in the United States and were interested in becoming a midwife to participate in the survey. Respondents were informed that the purpose of the study was to better understand the reasons they were exploring becoming a midwife and what in their life was supporting or hindering their ability to pursue midwifery education. We recruited respondents by posting the survey link on midwifery, childbirth, and reproductive justice listservs, social media platforms, and through emails to relevant midwifery and doula networks inviting them to further share the survey link with their constituencies. The project was deemed exempt from human subjects review by the institutional review board of UCSF (#20–33,105).

A total of 902 surveys were initiated (see Figure S1). Surveys were excluded if there were no recorded responses (*n* = 4) or if the email address or open‐ended response was duplicated (*n* = 9). Surveys were assessed for eligibility and were excluded if age (*n* = 9), race (*n* = 25), or residence (*n* = 10) were missing, or respondents did not identify as people of color (i.e., White non‐Latine or White and unknown ethnicity; *n* = 29) or were not currently residing in the United States (*n* = 17). Almost 90 percent of surveys that were initiated were included in the analysis. While comparative data on aspiring midwives of color are not available, a higher proportion of respondents identified as Black or African American, Indigenous, or more than one race, compared to a national sample of Certified Nurse‐Midwives and Certified Midwives of color.^23^ Respondents in this study had a younger age distribution, more diverse gender identities, and lower levels of income and education than Certified Nurse‐Midwives and Certified Midwives of all races.^23^

### 2.3 Measures and instrument

We used a socioecological framework^39^ to develop survey questions to assess the degree to which personal, familial, community, and societal factors influence individuals to pursue a career as a midwife. Personal factors included one's own experiences with pregnancy or discrimination in a health care setting. Familial factors included family members' health care experiences and careers. Community factors included community members' health care experiences and the presence of birth workers in the community. Societal factors included racial disparities in health care and maternal health outcomes. The survey included a total of 76 questions: 53 closed‐ended questions about motivators and barriers to pursuing a midwifery career; 12 closed‐ended questions about immediate intentions related to pursuing midwifery education; 10 demographic questions; and 1 open‐ended question: “Is there anything else you would like us to know about your interest in becoming a midwife or what would help you achieve your goal?” Closed‐ended questions on motivators and barriers typically used a 5‐point Likert scale ranging from 1 (*no effect*) to 5 (*very strong effect*). For questions on personal factors, a ‘not applicable’ option was added. Respondents self‐identified their race by choosing all races that applied. We categorized race as one race, more than one race, or other race specified by respondents (see Table 1). For respondents who indicated multiple races, we did not collect data on their primary identity or whether they considered themselves to be multiracial. Study data were collected and managed using REDCap.^40^ The survey was pilot tested for understandability and ease of use by six aspiring midwives of color.

**TABLE 1:** Demographic characteristics of respondents (*n* = 799)

Characteristic	Number	Percent
Gender identity		
Female	721	90.2
Genderqueer/gender nonconforming	35	4.4
Different identity^a^	5	0.6
Missing	38	4.8
Age (years)		
18–19	4	0.5
20–29	257	32.2
30–39	378	47.3
40–49	122	15.3
50–59	24	3.0
≥60	14	1.8
Race		
Asian and Native Hawaiian or other Pacific Islanders alone	18	2.3
Black or African Americans alone	534	66.8
Indigenous alone^b^	61	7.6
White Latine	17	2.1
Other races alone^c^	18	2.3
More than one race	151	18.9
Ethnicity		
Latine	161	20.2
Not Latine	582	72.8
Missing	56	7.0
Education		
High school degree or equivalent or less	25	3.1
Some colleges, no degree	237	29.7
Associate degree	82	10.3
Bachelor's degree	301	37.7
Graduate degree	150	18.8
Missing	4	0.5
Income (individual)		
$0 to $19,999	263	32.9
$20,000 to $39,999	202	25.3
$40,000 to $59,999	150	18.8
$60,000 to $79,999	76	9.5
$80,000 to $99,999	34	4.3
≥$100,000	33	4.1
Missing	41	5.1
Residence		
Urban	398	49.8
Suburban	311	38.9
Rural	82	10.3
Missing	8	1.0
Region^d^		
Midwest	110	13.8
Northeast	142	17.8
South	348	43.6
West	192	24.0
Other	7	0.9

*Note*: Missing encompasses the following responses: prefer not to answer, do not know, and no response.

^a^ Different identity includes non‐binary woman, femme, and gender questioning.

^b^ Indigenous includes American Indian or Native American people of North, South, or Central America.

^c^ Other race includes Arab, Hebrew, and other race not specified.

^d^ Regions based on United States Census Bureau regions; other includes, Puerto Rico, United States Virgin Islands, and more than one state.

### 2.4 Data collection

Respondents signed an electronic informed consent before initiating the survey. Upon completion of the survey, respondents had the opportunity to enter a raffle to receive one of 10 $25 gift cards. A link to an exclusive video on the history and contributions of Black midwives from Jennie Joseph at Commonsense Childbirth Inc. was provided to thank respondents who completed the survey. To maintain confidentiality, the survey was anonymous, however, respondents who volunteered to participate in interviews provided their email addresses.

### 2.5 Analysis

We calculated descriptive statistics and bivariate analyses (chi‐square and Fisher's exact tests where appropriate) to examine differences in survey responses, which may vary by demographic characteristics,^41^ using SAS software (version 9.4, SAS Institute Inc., Cary, NC, USA). In bivariate analyses, survey responses were dichotomized as very strong effect or not. We used a Bonferroni corrected *p*‐value of 0.00031 based on 159 bivariate analyses. One author open‐coded responses from the open‐ended question using Dedoose (Version: 9.0.17. SocioCultural Research Consultants, LLC, Los Angeles, CA). We then met to discuss and reach a consensus on the codes and categorization of codes, and identified exemplar quotes that illustrated findings from the descriptive data.

## 3 RESULTS

Demographic characteristics of 799 eligible respondents included in the analysis are shown in Table 1. Nine out of 10 respondents were female (90.2 percent) and 4.4 percent were genderqueer or gender non‐conforming. About two‐thirds of respondents were 30 years or older (67.3 percent) and a similar proportion identified as Black or African American alone (66.8 percent). Almost two out of 10 respondents identified as more than one race (18.9 percent). A more detailed description of respondents' race and ethnicity is shown in Table S1. Over half of respondents (56.4 percent) had a bachelor's degree or higher. More than half (58.2 percent) reported an annual individual income of less than $40,000. Of those with a bachelor's degree or higher, 27.7 percent had an income of less than $40,000. Respondents resided in every state except Montana, New Hampshire, and Vermont, as well as in Puerto Rico, and the United States Virgin Islands. Almost half of the respondents lived in an urban location (49.8 percent).

### 3.1 Key findings

#### 3.1.1 Motivators for becoming a midwife

**FIGURE 1:**
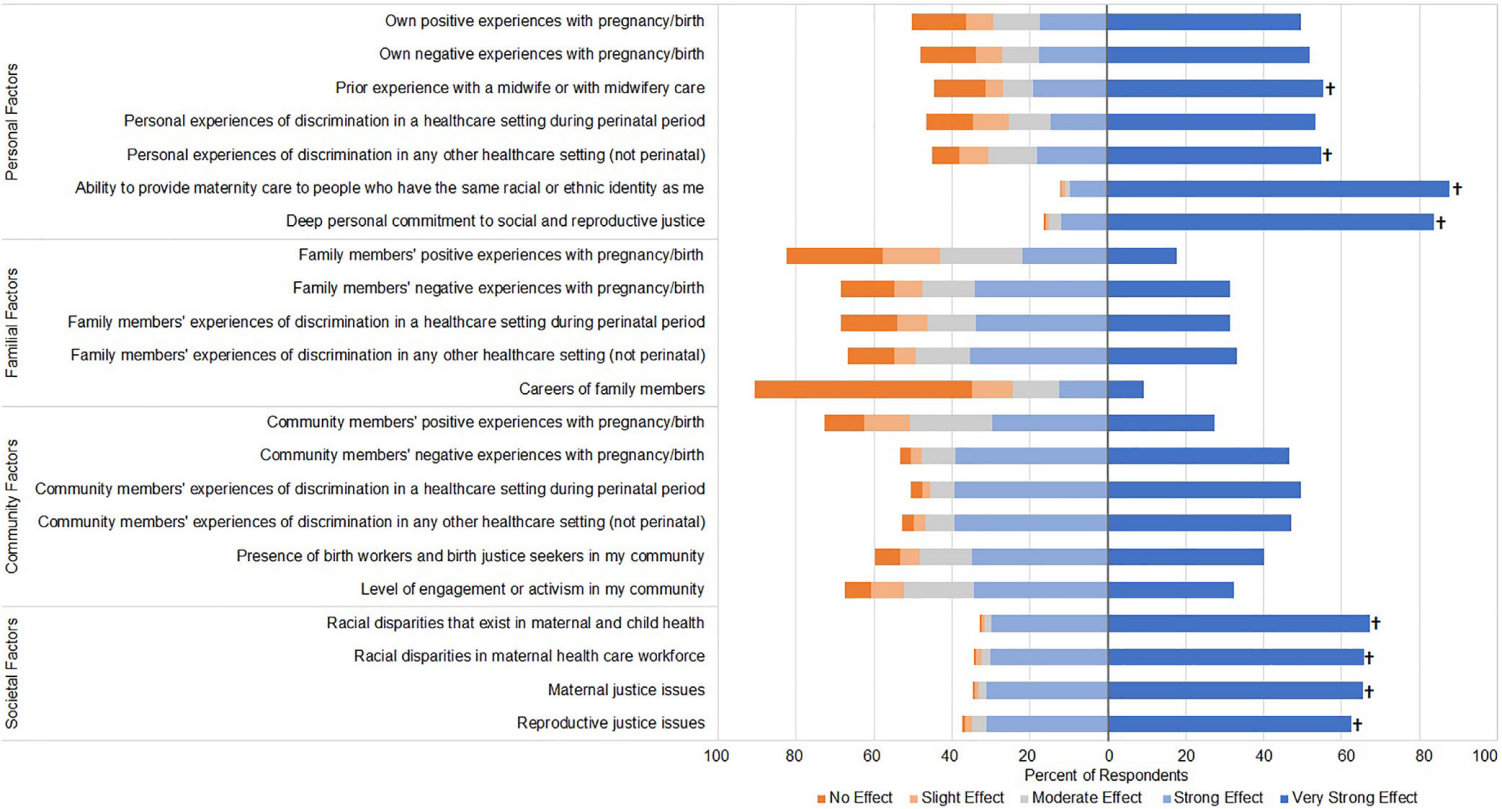
Level of effect of motivating factors on wanting to become a midwife (*n* = 799). † Indicates factors in top tertile of percent of respondents who indicated that the factor had a very strong effect on their desire to become a midwife. Respondents could respond “Not applicable” to personal factors and if so, were not included in this figure. [Color figure can be viewed at wileyonlinelibrary.com]

A high percentage of respondents reported personal and societal factors had a very strong effect on their desire to become a midwife (see Figure 1). The highest rated motivating factor for becoming a midwife was the ability to provide maternity care to people who have the same racial or ethnic identity as themselves, with 87.7 percent of respondents reporting this as a very strong motivating factor. Having a deep personal commitment to social and reproductive justice was also a very strong motivating factor for 83.6 percent of respondents. In their comments, respondents expressed their desire to provide birthing people‐centered and traditional midwifery care to improve outcomes among underserved populations who have the same identity as themselves, including Black, Indigenous, genderqueer, and trans birthing people. A multiracial woman in her 20s from the West wrote: *“As a mother, a Black woman and a doula, becoming a midwife is something I feel is necessary for not only myself, but my community and my lineage. I feel strongly about traditional midwifery where the mother is in control of her birth. Where birth isn't something she feels disconnected to. Women and birthing people should feel empowered as they usher in new life. Birthing should feel supported and joyous. I feel like western medicine has taken that away from birthing and birth is now handled with lots of fear masked as prevention. I want to provide mothers with internal resources to navigate birthing. I want Black mothers to deal with less trauma around birthing and have healthy pregnancies, babies and motherhood.”* See Table 2 for additional exemplary quotes related to motivators and barriers to becoming a midwife.

**TABLE 2:** Exemplary quotes of motivators and barriers to becoming a midwife from open‐ended survey question

Factors	Topic	Quotes
Motivators	Racially concordant care	*“I have been a doula for the past 3 years and have attended over 30 births. My purpose is to serve the Immigrant and Indigenous community. My family is from Oaxaca Mexico and I aspire to honor and represent traditional and natural ways of birthing. I wish to empower the community to reclaim ancestral healing through birthwork. I'm the first in my family to graduate from college and a daughter of immigrant Indigenous parents, I am walking a path that's been paved by my ancestors also being the first in my family, it is intimating and imposter syndrome is real!”* (Indigenous Latine woman in her 20s from the West) *“Queer and trans midwifery is important to me! This is a highly underserved population even within the Black community. Not enough practices are inclusive let alone queer centric ‐ I find that I always have to silence being with Black or Queer in order to navigate the birthkeeping world and it's discouraging.”* (Genderqueer/gender nonconforming, multiracial Latine in their 30s from the Midwest)
	Maternal and reproductive justice	*“I see the mortality rate in black women, I want to be a part of that change.”* (Black woman in her 50s from the South) *“Three factors that have given me a greater interest in midwifery was the disadvantages as a black mother in America, the lack of opportunities for people who are LGBTQIA and/or have a disability (mute, blind or deaf).”* (Black woman in her 20s from the South)
Barriers	Financial barriers	*“Scholarships would be a huge help or even paid internships to get experience and earn wages at the same time. I have always wanted to serve families but I will not be able to afford school full time with no incoming money.”* (Black woman in her 20s from the South) *“No Certified Professional Midwife program is supportive for the person that must work full time to support themselves. I am single, yes, but I pay all my own bill's, some of my family's (mother, niece, grandma) [bills], and prior educational loans. Since this is my second degree or program, I pay out of pocket. I cannot do that and not work. I really wish my program was work at your own pace. There needs to be an option for a work at your own pace for Bachelors (sic) of Midwifery. This is what will help minority women succeed.”* (Black woman in her 30s from the South) *“I am already enrolled in school and I am in the middle of my first year. The hardest part of the process was finding funding and now it is choosing to stay in a program that has so many structural issues within it. Having mentors tell me that midwifery education (for Certified Midwives and Certified Licensed Midwives) ‘sucks’ everywhere is very disheartening. We already lost 1/4 of our cohort because how our program is structured and not able to support people of Color through this process.”* (Indigenous woman in her 30s from the West)
	Lack of midwifery educators and mentors who are people of color	*“It's not just important to have a Black OWNED midwifery school, but also have a Black midwifery school with Black teachers/professors.”* (Black woman in her 40s from the Northeast) *“I'm currently in a traditional midwifery school, it is a good program but it's run by a White woman, so I do feel that as a Black birth worker I am lacking in so much ancestral and cultural info that I would receive working with birthkeepers that look like me.”* (Black Latine woman in her 20s from the South)
	Lack of preceptors and apprenticeships for people of color	*“Preceptorship and finding a midwife to practice with that have enough births in my area is a barrier. There are limited midwifery services and I work as a midwife assistant, she does not want to be my preceptor though and I do not get called to enough births. I'm willing to travel to attend under a midwife, but cannot move. Midwifery feels like a locked glass door.”* (Black woman in her 30s from the Northeast) *“I'm looking to study and though my area is saturated with midwifes and birth centers there aren't many midwifes of color or ones that are preceptors.”* (Black woman in her 20s from the South) *“I have reached out and tried reaching out to White midwives in my area to see if I can study under them, but I get no response. We only have two midwives in the area and I do not want to relocate, because I am a single mom and my family support is here.”* (Black woman in her 20s from the South) *“The schooling is hard to get in without the money and either already college credit. Finding apprenticeship is even harder. It's as if someone wants to keep certain people out of the industry.”* (Black woman in her 30s from the West)

Approximately two‐thirds of respondents reported societal factors, such as racial disparities in maternal and child health (67.2 percent) and the maternal health care workforce (65.7 percent), and maternal (65.4 percent) and reproductive (62.6 percent) justice issues, were very strong motivating factors (see Figure 1). In their comments, respondents described urgency in wanting to become a midwife to address racial disparities in maternal health as a result of systemic racism. A Black woman in her 20s from the Midwest wrote: *“My mother passed away in childbirth with me because of systemic racism. The way that I played as a child and my passion for learning about birth is my purpose. I feel this work so deeply and understand that we cannot wait any longer.”*

Over half of respondents reported personal factors such as a prior experience with a midwife (55.4 percent) and personal experiences of discrimination in a health care setting not during the perinatal period (54.8 percent) as very strong motivating factors. While not among the strongest motivating factors, experiences of discrimination in health care settings among community members were also important motivating factors (49.5 percent reported experiences of discrimination during the perinatal period and 47.2 percent reported experiences of discrimination not during the perinatal period as strong motivating factors). Respondents described how they desired to provide a safe space for birthing people of color, in part to overcome the mistreatment communities of color experience in health care settings and resulting distrust of White health care providers. A Black woman in her 30s from the South wrote: *“Through my journey I have come across so many women in my community with horror stories about their hospital experiences and yet were too afraid to step outside the box because no one looked like them. I want to bridge the gap for my sisters who may feel distrustful of a midwife who doesn't look like them even though they may be great. There is a lot of mistrust of people who are not Black/or brown in my community and I would like to be a safe place for my sisters.”* The fewest respondents (9.3 percent) reported careers of family members as a very strong motivating factor.

#### 3.1.2 Barriers to becoming a midwife

**FIGURE 2:**
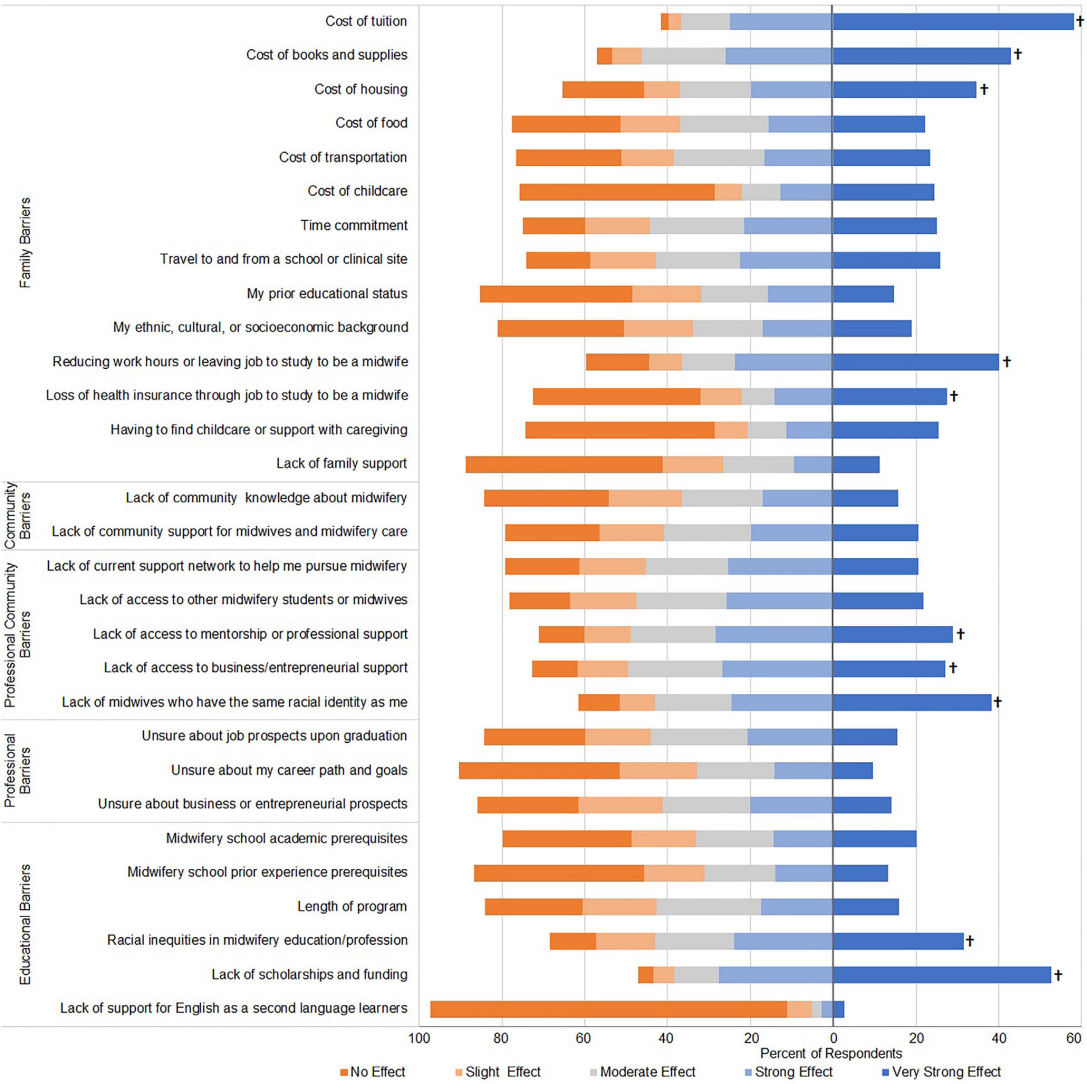
Level of effect of barriers on decision or ability to become a midwife (*n* = 799). † Indicates factors in top tertile of percent of respondents who indicated that the barrier had a very strong effect on their decision or ability to become a midwife. [Color figure can be viewed at wileyonlinelibrary.com]

A high percentage of respondents reported personal financial factors and educational and professional community factors were barriers to becoming a midwife (Figure 2). Leading financial barriers to becoming a midwife were the cost of tuition (58.2 percent), lack of scholarships and funding (52.8 percent), cost of books and supplies (43.0 percent), reduced work hours to study to be a midwife (40.2 percent), cost of housing (34.7 percent), and loss of health insurance if they had to quit work or reduce hours to participate in midwifery education (27.5 percent). In their comments, respondents explained how they had desired to become a midwife for years, however, financial barriers delayed or prevented them from entering and remaining in a midwifery education program. A Cuban/Latine woman in her 30s from the South wrote: *“I feel the barrier to entry really boils down to the massive financial commit, with no opportunity to utilize state grants, financial aid or scholarships.”*

Almost one‐third of respondents reported racial inequities in midwifery education and the profession of midwifery (31.5 percent) as being a very strong barrier to entry into a midwifery program. Respondents also reported professional community factors were barriers, including a lack of midwives with the same racial identity as respondents (38.4 percent), lack of mentorship (29.0 percent), and lack of business or entrepreneurial support (27.2 percent). Respondents described a lack of support for midwifery students of color and expressed the importance of having Black‐owned midwifery schools, Black teachers, and learning about ancestral and cultural teachings of Black midwifery educators and mentors. A Black woman in her 30s from the Northeast commented: *“I have been on this path for years. Just signed up to finish. I have to advocate for myself, my school wasn't backing up the support they said they had for Black student midwives. So, moving forward I feel like I will always have to ‘fight’ to become a midwife. I have to fight to save my people. That is just crazy to me.”*

Respondents described a lack of clinical preceptors with whom to apprentice near to where they lived, and specifically a lack of preceptors of color, as a barrier to becoming a midwife. Respondents felt existing midwives were gatekeepers of the profession and respondents' perceived racism was the reason they were unable to find a midwife with whom to do an apprenticeship. A Black woman in her 30s from the Midwest wrote: *“Support in my area would be nice. No local midwives so far have wanted to do an apprenticeship with me. The only thing (sic) I could think is the color of my skin.”* Respondents also expressed a lack of midwifery programs as an important barrier to becoming a midwife. A Black woman in her 30s from the Northeast wrote: *“There is always a waiting list to get into programs that you don't need the pre‐requisites like bio or chem. We need more programs and mentors for future midwives.”* The fewest respondents (2.7 percent) reported lack of support for English as a second language learner as being a very strong barrier.

#### 3.1.3 Bivariate analyses by demographic characteristics

A higher percentage of respondents who identified as Black or African American alone (91.5 percent) or Indigenous alone (89.5 percent), than those who identified as White Latine (37.5 percent), indicated that the ability to provide maternity care to people who have the same racial or ethnic identity as me had a very strong effect on their desire to become a midwife (*p* < 0.0001, Table S2). A higher percentage of respondents with lower incomes, than those with higher incomes, indicated that cost of tuition (*p* < 0.0001) and child care (*p* = 0.0003) had a very strong effect as a barrier (Table S3). Whereas, a higher percentage of respondents with higher incomes, than those with lower incomes, indicated that reducing work hours or leaving a job to study to be a midwife (*p* < 0.0001) and loss of health insurance through a job to study to be a midwife (*p* < 0.0001) had a very strong effect as a barrier. A higher percentage of respondents with a lower level of education, than those with a higher level of education, indicated that barriers such as cost of books and supplies (*p* < 0.0001), prior education status (*p* < 0.0001), ethnic, cultural or socioeconomic background (*p* < 0.0001), and midwifery school academic prerequisites (*p* < 0.0001) had a very strong effect as a barrier (Table S4).

## 4 DISCUSSION

Results of this survey provide the first national description of motivators and barriers to entry into midwifery education and the midwifery profession by people of color. Our findings indicate structural and interpersonal racisms are both motivators and barriers for aspiring midwives of color. Providing racially concordant care for community members and reducing racial disparities in health are among the strongest motivating factors for aspiring midwives of color. Furthermore, personal experiences related to midwifery care and health care are important motivating factors. Main barriers to entering midwifery are direct and related costs of midwifery education and lack of racial concordance in midwifery education and the midwifery profession that may contribute to racially motivated exclusion.

Much of the limited existing research on what motivates people of color to become midwives come from qualitative studies. Our findings on motivators, such as providing racially concordant care and a commitment to social and reproductive justice, are consistent with these studies.^33^ These motivating factors are similar for other birth workers of color, such as doulas.^34^ Our findings that both positive and negative personal birth experiences are motivating factors are consistent with motivating factors among mostly White people entering midwifery.^35^, ^42^ In contrast to one qualitative study among mostly midwives of color which found one‐third of midwives were descendants of midwives,^37^ we found in our large national study that family career path was ranked the lowest motivator. This finding speaks to the systematic eradication of midwives of color throughout the 19th and 20th centuries by the medical profession, public health officials, and modern‐day nurse‐midwifery. Black and Indigenous midwives in the US played an important role in their communities while providing a measure of agency and control in an environment where both had been forcibly taken through a long legacy of forced removal and forced labor.^43^, ^44^, ^45^ Federal programs, first with the Sheppard‐Towner Maternity and Infancy Protection Act of 1921 and later with the Hill‐Burton Act of 1946, divided midwifery along racial and educational lines and efficiently ended the long tradition of midwives of color safely serving their communities.^44^ Current aspiring midwives may be several generations removed from their grand midwife ancestors and uplifting this important history and heritage may be crucial to the recruitment and retention of a new generation of midwives of color.

To our knowledge, our study is the first to examine a wide range of barriers to entering midwifery education among people of color. We found costs of midwifery education were a major barrier. It is striking that over half of respondents had a bachelor's degree and yet over a quarter of these respondents reported having an income of less than $40,000. This disparity in education and income may indicate that highly educated people of color are unable to find gainful employment or are undercompensated for their knowledge and skills, which then impedes their ability to pursue midwifery. Our findings are consistent with qualitative studies on barriers Black student midwives experience, including institutional and interpersonal racism in midwifery education, professional organizations, and clinical practice, particularly overt racism and lack of willing preceptors.^37^, ^38^

### 4.1 Strengths and limitations

We recruited a large and diverse national sample of people of color who aspire to become midwives. However, because of the recruitment strategy using social media and other public channels, we were unable to report non‐response bias. We conducted the survey in English, and therefore our results may not be generalizable to aspiring midwives of color for whom English is a second language. Our sample included people who desired to or were on the pathway to becoming a midwife, including some who indicated in their comments they were already enrolled in a midwifery program. Respondents already enrolled in midwifery education may have different barriers and motivators than those who have not yet enrolled or are still considering their next steps.

### 4.2 Implications

To address both structural and interpersonal racism, our findings support a national imperative to recruit and educate a diversified perinatal workforce as a matter of urgency to address the maternal health crisis in the US,^20^, ^46^, ^47^ especially in communities of color. Policy interventions to increase diversity in nursing and other health care workforces suggest providing funding to students of color,^48^, ^49^ providing culturally‐competent midwifery education,^50^, ^51^ creating a robust pipeline for midwives of color,^49^, ^50^ and opening more midwifery schools, especially in Historically Black Colleges and Universities,^52^ or through collaborations between community and state colleges and private and public entities. Multiple funding streams are needed to support enrollment and retention of students of color, particularly those with lower levels of income or education, including scholarships for tuition, books, child care, and other living costs; publicly and privately funded grants; federally‐backed low‐interest loans; and loan forgiveness programs for those who work in underserved areas.^48^, ^49^ Midwifery schools should redevelop their curriculum to address the interests of students of color in providing holistic care that incorporates traditional or ancestral midwifery practices. Midwifery schools that are committed to addressing racial disparities in health must create a robust pipeline that includes enrolling more students of color; training, mentoring, and paying preceptors of color for their labor; and hiring more faculty of color at all levels.^51^ Opening midwifery schools in Historically Black Colleges and Universities would be a logical step in attracting more Black students and providing a supportive educational environment for aspiring Black midwives to succeed.^52^ Additionally, support for midwives of color to become directors of midwifery educational and clinical programs, and owners of private businesses and private practices are also necessary to address racial disparities in health.

Future research should examine factors that predict enrollment and retention of midwifery students of color, retention of preceptors of color, and retention in the profession. Longitudinal studies are needed to better understand facilitators and barriers related to different educational pathways to midwifery and retention in the profession. Comparative studies are needed to evaluate the effectiveness of different interventions to address the barriers to midwifery education.

### 4.3 Conclusions

Structural and interpersonal racisms are both motivators and barriers for aspiring midwives of color. Providing racially concordant care in the community, reducing racial disparities in health, and experiencing discrimination in health care settings are among the strongest motivating factors for aspiring midwives of color. However, costs of midwifery education, lack of racial concordance in midwifery education and the midwifery profession, and racially motivated exclusion from the profession are major barriers. Generational harms and a legacy of discrimination have erased Black and Indigenous midwives and healers from their communities, which may have a significant impact on who pursues midwifery education and careers. The urgent diversification and inclusion of professional and academic midwifery leadership, as well as public health, medical, and institutional leadership that understands the impact of these inequities and the need for funding, must become a priority in order to address this drastic workforce deficit and to mitigate the alarming maternal health disparities among communities of color.

## Supporting information


**Appendix S1.** Supporting Information.
