# Supplementary material for: Racism is a motivator and a barrier for people of color aspiring to become midwives in the United States
Source: Health Serv Res. 2022 Jul 23;58(1):40–50. doi: 10.1111/1475-6773.14037 (PMC9836944; doi:10.1111/1475-6773.14037)
Supplement: Supplementary file 1 — Appendix S1. Supporting Information. [file HESR-58-40-s001.docx]

Appendix Figure 1. Flow Chart of Surveys

**Screening**

**Eligibility**

**Included**

Surveys excluded with reasons (*n* = 13)

Survey questions not answered: 4

Duplicate survey: 9

Surveys ineligible with reasons (*n* = 90)

Missing age: 9

Missing race: 25

Missing residence: 10

Not eligible by race: 29

Not eligible by residence: 17

Surveys initiated

(*n* = 902)

Surveys assessed for eligibility

(*n* = 889)

**Surveys included in the analysis**

**(*n* = 799; 89.9%^a^)**

^a^ Percentage of surveys assessed for eligibility

Note: Not eligible by race includes white non-Latine and white and unknown ethnicity.

Appendix Table 1. Detailed Race and Ethnicity of Respondents (*n* = 799)

| Race and ethnicity | Number | Percent |
| --- | --- | --- |
| Asian and Native Hawaiian or other Pacific Islander |  |  |
| Asian and Native Hawaiian or other Pacific Islander alone | 18 | 2.3 |
| Asian and Native Hawaiian or other Pacific Islander and Black or African American | 7 | 0.9 |
| Asian and Native Hawaiian or other Pacific Islander and Indigenous | 3 | 0.4 |
| Asian and Native Hawaiian or other Pacific Islander and white Latine | 7 | 0.9 |
| Black or African American |  |  |
| Black or African American alone | 534 | 66.8 |
| Black or African American and Indigenous | 36 | 4.5 |
| Black or African American and white Latine | 34 | 4.3 |
| Black or African American and other race | 5 | 0.6 |
| Indigenous^a^ |  |  |
| Indigenous alone | 61 | 7.6 |
| Indigenous and white Latine | 17 | 2.1 |
| Indigenous and other race | 3 | 0.4 |
| White |  |  |
| White Latine | 17 | 2.1 |
| White Latine and other race | 2 | 0.3 |
| Other race^b^ | 23 | 2.9 |
| Three or more races | 32 | 4.0 |

^a^ Indigenous includes American Indian or Native American people of North, South, or Central America.

^b^ Other race includes Arab, Hebrew, and other race not specified.

Appendix Table 2. Bivariate Analyses of Motivators and Barriers by Race

| Level | Factor/Barrier | Percent of respondents who indicated the factor/barrier had a very strong effect on their desire/ability to become a midwife^a^ | | | | | | | Chi-square *p*-value |
| --- | --- | --- | --- | --- | --- | --- | --- | --- | --- |
|  |  | Asian and Native Hawaiian or other Pacific Islander alone | Black or African American alone | Indigenous alone^b^ | White Latine | Other race alone^c^ | More than one race | Total |  |
| MOTIVATORS | | | | | | | | | |
| Personal factors | Own positive experiences with pregnancy/birth | 66.7 | 47.0 | 54.4 | 28.6 | 50.0 | 57.0 | 49.6 | 0.169 |
|  | Own negative experiences with pregnancy/birth | 36.4 | 52.4 | 54.4 | 53.3 | 50.0 | 50.9 | 51.8 | 0.936 |
|  | Prior experience with a midwife or with midwifery care | 62.5 | 53.4 | 69.1 | 46.2 | 46.7 | 56.3 | 55.3 | 0.303 |
|  | Personal experiences of discrimination in a healthcare setting during perinatal period | 30.0 | 54.9 | 60.9 | 28.6 | 64.3 | 48.7 | 53.2 | 0.120 |
|  | Personal experiences of discrimination in any other healthcare setting (not perinatal) | 43.8 | 55.6 | 69.8 | 21.4 | 43.8 | 52.1 | 54.7 | 0.021 |
|  | **Ability to provide maternity care to people who have the same racial or ethnic identity as me** | **88.2** | **91.5** | **89.5** | **37.5** | **88.2** | **79.1** | **87.7** | **<0.0001^d^** |
| Personal factors | Deep personal commitment to social and reproductive justice | 81.3 | 82.8 | 91.4 | 68.8 | 75.0 | 86.5 | 83.6 | 0.161^d^ |
| Familial factors | Family members' positive experiences with pregnancy/birth | 22.2 | 18.1 | 25.0 | 18.8 | 17.7 | 12.6 | 17.6 | 0.330^d^ |
|  | Family members' negative experiences with pregnancy/birth | 16.7 | 32.5 | 39.3 | 18.8 | 23.5 | 28.7 | 31.4 | 0.297 |
|  | Family members' experiences of discrimination in a healthcare setting during perinatal period | 27.8 | 32.1 | 41.0 | 12.5 | 23.5 | 27.7 | 31.3 | 0.225 |
|  | Family members' experiences of discrimination in any other healthcare setting (not perinatal) | 41.2 | 32.7 | 48.3 | 12.5 | 35.3 | 30.2 | 33.2 | 0.063 |
|  | Careers of family members | 5.6 | 10.5 | 4.9 | 6.3 | 0.0 | 8.7 | 9.3 | 0.624^d^ |
| Community factors | Community members' positive experiences with pregnancy/birth | 50.0 | 27.2 | 26.7 | 31.3 | 11.8 | 26.5 | 27.2 | 0.247^d^ |
|  | Community members' negative experiences with pregnancy/birth | 55.6 | 46.3 | 50.8 | 43.8 | 17.7 | 48.7 | 46.6 | 0.214 |
|  | Community members' experiences of discrimination in a healthcare setting during perinatal period | 55.6 | 50.2 | 53.3 | 37.5 | 35.3 | 47.7 | 49.5 | 0.668 |
|  | Community members' experiences of discrimination in any other healthcare setting (not perinatal) | 50.0 | 48.1 | 55.0 | 25.0 | 35.3 | 44.4 | 47.2 | 0.277 |
|  | Presence of birth workers and birth justice seekers in my community | 55.6 | 41.5 | 39.3 | 31.3 | 23.5 | 36.7 | 40.1 | 0.362 |
|  | Level of engagement or activism in my community | 55.6 | 32.1 | 33.3 | 37.5 | 23.5 | 31.1 | 32.4 | 0.375 |
| Societal factors | Racial disparities that exist in maternal and child health | 72.2 | 70.5 | 60.0 | 37.5 | 58.8 | 61.8 | 67.1 | 0.022 |
|  | Racial disparities in maternal health care workforce | 55.6 | 69.5 | 58.3 | 43.8 | 58.8 | 59.9 | 65.7 | 0.039 |
|  | Maternal justice issues | 66.7 | 68.1 | 60.7 | 50.0 | 64.7 | 59.6 | 65.4 | 0.288 |
|  | Reproductive justice issues | 66.7 | 63.7 | 56.7 | 43.8 | 64.7 | 62.5 | 62.6 | 0.587 |
| BARRIERS | | | | | | | | | |
| Personal barriers | Cost of tuition | 50.0 | 57.4 | 67.2 | 43.8 | 64.7 | 59.2 | 58.2 | 0.494 |
|  | Cost of books and supplies | 22.2 | 42.2 | 50.8 | 26.7 | 41.2 | 47.0 | 42.9 | 0.186 |
|  | Cost of housing | 22.2 | 31.9 | 41.7 | 25.0 | 35.3 | 44.1 | 34.6 | 0.053 |
|  | Cost of food | 16.7 | 19.5 | 35.0 | 12.5 | 29.4 | 28.0 | 22.3 | 0.031^d^ |
|  | Cost of transportation | 22.2 | 19.6 | 34.4 | 18.8 | 29.4 | 32.2 | 23.4 | 0.007^d^ |
|  | Cost of childcare | 22.2 | 21.7 | 35.6 | 31.3 | 41.2 | 26.9 | 24.3 | 0.079^d^ |
|  | Time commitment | 27.8 | 24.9 | 24.6 | 31.3 | 35.3 | 24.0 | 25.1 | 0.880^d^ |
|  | Travel to and from a school or clinical site | 33.3 | 24.3 | 36.1 | 18.8 | 23.5 | 28.0 | 25.9 | 0.371^d^ |
|  | My prior educational status | 5.6 | 14.6 | 18.0 | 18.8 | 11.8 | 14.6 | 14.6 | 0.851^d^ |
| Personal barriers | My ethnic, cultural, or socioeconomic background | 11.1 | 18.5 | 26.7 | 12.5 | 17.7 | 19.3 | 18.9 | 0.666^d^ |
|  | Reducing work hours or leaving job to study to be a midwife | 27.8 | 40.6 | 52.5 | 31.3 | 43.8 | 35.5 | 40.1 | 0.220 |
|  | Loss of health insurance through job to study to be a midwife | 16.7 | 27.0 | 37.7 | 31.3 | 29.4 | 25.8 | 27.5 | 0.466^d^ |
| Family barriers | Having to find childcare or support with caregiving | 16.7 | 21.9 | 38.3 | 31.3 | 52.9 | 30.5 | 25.5 | 0.003^d^ |
|  | Lack of family support | 11.1 | 9.5 | 15.0 | 13.3 | 12.5 | 16.0 | 11.2 | 0.214^d^ |
| Community barriers | Lack of community knowledge about midwifery | 16.7 | 14.6 | 19.7 | 20.0 | 11.8 | 17.9 | 15.7 | 0.764^d^ |
|  | Lack of community support for midwives and midwifery care | 22.2 | 20.3 | 21.7 | 18.8 | 23.5 | 21.2 | 20.6 | 0.994^d^ |
| Professional community barriers | Lack of current support network to help me pursue midwifery | 22.2 | 20.3 | 35.0 | 18.8 | 29.4 | 15.1 | 20.6 | 0.046^d^ |
| Professional community barriers | Lack of access to other midwifery students or midwives | 22.2 | 20.9 | 31.2 | 12.5 | 29.4 | 21.1 | 21.7 | 0.441^d^ |
|  | Lack of access to mentorship or professional support | 22.2 | 28.2 | 34.4 | 18.8 | 41.2 | 30.5 | 29.0 | 0.599 |
|  | Lack of access to business/entrepreneurial support | 33.3 | 26.6 | 23.7 | 20.0 | 29.4 | 30.3 | 27.1 | 0.854^d^ |
|  | Lack of midwives who have the same racial identity as me | 55.6 | 38.1 | 43.3 | 18.8 | 35.3 | 38.2 | 38.4 | 0.351 |
| Professional barriers | Unsure about job prospects upon graduation | 16.7 | 16.5 | 8.3 | 18.8 | 11.8 | 15.2 | 15.6 | 0.671^d^ |
|  | Unsure about my career path and goals | 11.1 | 9.7 | 4.9 | 18.8 | 5.9 | 10.7 | 9.6 | 0.556^d^ |
|  | Unsure about business or entrepreneurial prospects | 23.5 | 14.4 | 13.1 | 12.5 | 11.8 | 13.2 | 14.1 | 0.892^d^ |
| Educational barriers | Midwifery school academic prerequisites | 16.7 | 20.7 | 24.6 | 12.5 | 17.7 | 18.7 | 20.2 | 0.915^d^ |
| Educational barriers | Midwifery school prior experience prerequisites | 22.2 | 12.8 | 13.3 | 6.3 | 23.5 | 13.3 | 13.2 | 0.576^d^ |
|  | Length of program | 33.3 | 16.6 | 21.3 | 6.3 | 17.7 | 9.9 | 15.8 | 0.049^d^ |
|  | Racial inequities in midwifery education/profession | 47.1 | 31.1 | 42.6 | 6.3 | 29.4 | 29.3 | 31.4 | 0.061 |
|  | Lack of scholarships and funding | 44.4 | 51.3 | 65.6 | 37.5 | 52.9 | 55.7 | 52.8 | 0.223 |
|  | Lack of support for English as a second language learners | 5.9 | 2.5 | 1.6 | 12.5 | 5.9 | 2.0 | 2.7 | 0.121^d^ |

^a^ Survey responses were dichotomized as very strong effect or not (i.e., no, slight, moderate, and strong effect).

^b^ Indigenous includes American Indian or Native American people of North, South, or Central America.

^c^ Other race includes Arab, Hebrew, and other race not specified.

^d^ *P*-value from Fisher’s exact test.

Note: Bolded entries are significant based on a Bonferroni corrected *p*-value of 0.00031.

Appendix Table 3. Bivariate Analyses of Motivators and Barriers by Income

| Level | Factor/Barrier | Percent of respondents who indicated the factor/barrier had a very strong effect on their desire/ability to become a midwife^a^ | | | | | | Chi-square *p*-value |
| --- | --- | --- | --- | --- | --- | --- | --- | --- |
|  |  | $0 - $19,999 | $20,000 - $39,999 | $40,000 - $59,999 | $60,000 - $79,999 | >$80,000 | Total |  |
| MOTIVATORS | | | | | | | | |
| Personal factors | Own positive experiences with pregnancy/birth | 50.5 | 52.3 | 51.6 | 45.3 | 38.8 | 49.6 | 0.502 |
|  | Own negative experiences with pregnancy/birth | 54.4 | 52.5 | 55.5 | 50.0 | 45.8 | 52.9 | 0.817 |
|  | Prior experience with a midwife or with midwifery care | 57.6 | 57.3 | 48.6 | 49.1 | 60.0 | 55.2 | 0.417 |
|  | Personal experiences of discrimination in a healthcare setting during perinatal period | 54.9 | 55.1 | 47.1 | 56.6 | 50.0 | 53.2 | 0.660 |
|  | Personal experiences of discrimination in any other healthcare setting (not perinatal) | 55.8 | 53.8 | 53.6 | 52.9 | 62.7 | 55.2 | 0.771 |
|  | Ability to provide maternity care to people who have the same racial or ethnic identity as me | 84.8 | 88.9 | 92.5 | 89.5 | 85.9 | 88.0 | 0.204 |
|  | Deep personal commitment to social and reproductive justice | 82.7 | 83.3 | 88.1 | 84.0 | 80.0 | 83.8 | 0.571 |
| Familial factors | Family members' positive experiences with pregnancy/birth | 15.1 | 19.9 | 22.2 | 17.1 | 11.9 | 17.7 | 0.247 |
|  | Family members' negative experiences with pregnancy/birth | 30.7 | 34.2 | 37.6 | 26.3 | 22.4 | 31.8 | 0.149 |
|  | Family members' experiences of discrimination in a healthcare setting during perinatal period | 27.8 | 36.1 | 36.7 | 29.0 | 24.2 | 31.6 | 0.119 |
|  | Family members' experiences of discrimination in any other healthcare setting (not perinatal) | 30.9 | 36.2 | 37.8 | 35.5 | 23.9 | 33.5 | 0.231 |
|  | Careers of family members | 6.2 | 13.5 | 9.7 | 10.8 | 6.0 | 9.3 | 0.083 |
| Community factors | Community members' positive experiences with pregnancy/birth | 26.4 | 30.4 | 32.7 | 19.7 | 17.9 | 27.3 | 0.078 |
|  | Community members' negative experiences with pregnancy/birth | 48.9 | 49.0 | 51.3 | 39.5 | 29.9 | 46.8 | 0.022 |
|  | Community members' experiences of discrimination in a healthcare setting during perinatal period | 49.8 | 53.5 | 54.7 | 43.4 | 34.9 | 49.8 | 0.047 |
| Community factors | Community members' experiences of discrimination in any other healthcare setting (not perinatal) | 45.2 | 51.3 | 54.0 | 40.8 | 37.3 | 47.4 | 0.079 |
|  | Presence of birth workers and birth justice seekers in my community | 39.0 | 46.8 | 42.0 | 36.8 | 28.4 | 40.5 | 0.085 |
|  | Level of engagement or activism in my community | 35.5 | 36.2 | 38.5 | 21.1 | 15.2 | 33.0 | 0.001 |
| Societal factors | Racial disparities that exist in maternal and child health | 67.7 | 66.8 | 70.5 | 67.1 | 59.7 | 67.2 | 0.649 |
|  | Racial disparities in maternal health care workforce | 66.5 | 63.9 | 67.1 | 67.1 | 62.1 | 65.6 | 0.917 |
|  | Maternal justice issues | 66.7 | 66.7 | 68.0 | 61.8 | 62.1 | 66.1 | 0.846 |
|  | Reproductive justice issues | 65.4 | 65.7 | 65.3 | 54.7 | 52.2 | 63.2 | 0.127 |
| BARRIERS | | | | | | | | |
| Personal barriers | **Cost of tuition** | **65.5** | **63.4** | **54.0** | **51.3** | **34.9** | **58.5** | **<0.0001** |
|  | Cost of books and supplies | 48.5 | 49.3 | 38.1 | 34.2 | 29.9 | 43.5 | 0.005 |
|  | Cost of housing | 38.9 | 39.5 | 33.1 | 27.6 | 23.9 | 35.5 | 0.058 |
| Personal barriers | Cost of food | 26.4 | 24.4 | 22.8 | 14.9 | 14.9 | 23.0 | 0.131 |
|  | Cost of transportation | 31.3 | 24.9 | 18.9 | 11.8 | 17.9 | 24.0 | 0.002 |
|  | **Cost of childcare** | **32.8** | **27.3** | **20.3** | **9.3** | **19.4** | **25.3** | **0.0003** |
|  | Time commitment | 28.8 | 22.3 | 26.9 | 19.7 | 20.9 | 25.0 | 0.313 |
|  | Travel to and from a school or clinical site | 31.8 | 19.4 | 35.1 | 17.1 | 22.4 | 26.8 | 0.001 |
|  | My prior educational status | 14.6 | 15.4 | 16.3 | 7.9 | 16.4 | 14.6 | 0.500 |
|  | My ethnic, cultural, or socioeconomic background | 21.5 | 22.5 | 17.3 | 10.5 | 12.5 | 19.0 | 0.085 |
|  | **Reducing work hours or leaving job to study to be a midwife** | **26.8** | **41.6** | **51.3** | **56.6** | **43.3** | **40.1** | **<0.0001** |
|  | **Loss of health insurance through job to study to be a midwife** | **17.7** | **26.7** | **34.7** | **42.1** | **40.3** | **28.0** | **<0.0001** |
| Family barriers | Having to find childcare or support with caregiving | 32.8 | 25.6 | 18.7 | 21.1 | 24.2 | 26.1 | 0.022 |
|  | Lack of family support | 14.7 | 10.6 | 10.0 | 9.2 | 12.3 | 11.9 | 0.504 |
| Community barriers | Lack of community knowledge about midwifery | 22.7 | 13.4 | 13.3 | 11.8 | 9.0 | 16.0 | 0.008 |
|  | Lack of community support for midwives and midwifery care | 26.9 | 18.5 | 18.7 | 14.7 | 16.4 | 20.9 | 0.051 |
| Professional community barriers | Lack of current support network to help me pursue midwifery | 23.0 | 19.4 | 23.3 | 17.6 | 16.4 | 21.0 | 0.592 |
|  | Lack of access to other midwifery students or midwives | 28.6 | 17.4 | 22.3 | 17.3 | 16.4 | 22.2 | 0.024 |
|  | Lack of access to mentorship or professional support | 34.9 | 22.3 | 29.3 | 36.0 | 20.9 | 29.3 | 0.013 |
|  | Lack of access to business/entrepreneurial support | 31.0 | 21.6 | 30.4 | 26.7 | 26.9 | 27.6 | 0.219 |
|  | Lack of midwives who have the same racial identity as me | 43.9 | 36.3 | 39.3 | 37.3 | 29.9 | 39.1 | 0.223 |
| Professional barriers | Unsure about job prospects upon graduation | 13.9 | 10.5 | 20.3 | 22.7 | 19.4 | 15.6 | 0.032 |
|  | Unsure about my career path and goals | 8.1 | 6.5 | 11.6 | 16.0 | 9.0 | 9.2 | 0.125 |
|  | Unsure about business or entrepreneurial prospects | 11.5 | 12.0 | 17.6 | 20.0 | 15.2 | 14.0 | 0.211 |
| Educational barriers | Midwifery school academic prerequisites | 24.0 | 21.4 | 15.4 | 19.2 | 16.4 | 20.5 | 0.269 |
|  | Midwifery school prior experience prerequisites | 15.7 | 12.5 | 14.1 | 8.1 | 9.0 | 13.2 | 0.360 |
|  | Length of program | 15.3 | 12.0 | 16.1 | 21.3 | 23.9 | 15.9 | 0.126 |
| Educational barriers | Racial inequities in midwifery education/profession | 36.1 | 29.0 | 28.6 | 26.7 | 27.7 | 31.0 | 0.304 |
|  | Lack of scholarships and funding | 57.5 | 56.7 | 48.7 | 50.7 | 37.9 | 53.1 | 0.032 |
|  | Lack of support for English as a second language learners | 2.3 | 2.5 | 4.8 | 1.4 | 3.1 | 2.8 | 0.609^b^ |

^a^ Survey responses were dichotomized as very strong effect or not (i.e., no, slight, moderate, and strong effect).

^b^ *P*-value from Fisher’s exact test.

Note: Bolded entries are significant based on a Bonferroni corrected *p*-value of 0.00031.

Appendix Table 4. Bivariate Analyses of Motivators and Barriers by Education Level

| Level | Factor/Barrier | Percent of respondents who indicated the factor/barrier had a very strong effect on their desire/ability to become a midwife^a^ | | | | | Chi-square *p*-value |
| --- | --- | --- | --- | --- | --- | --- | --- |
|  |  | High school degree or equivalent or less | Some college, no degree | Associate degree | Bachelor's or graduate degree | Total |  |
| MOTIVATORS | | | | | | | |
| Personal factors | Own positive experiences with pregnancy/birth | 38.9 | 49.7 | 55.2 | 49.2 | 49.7 | 0.638 |
|  | Own negative experiences with pregnancy/birth | 52.6 | 61.8 | 51.6 | 45.7 | 52.2 | 0.008 |
|  | Prior experience with a midwife or with midwifery care | 66.7 | 58.0 | 55.2 | 53.4 | 55.3 | 0.624 |
|  | Personal experiences of discrimination in a healthcare setting during perinatal period | 57.9 | 59.7 | 58.5 | 47.1 | 53.0 | 0.037 |
|  | Personal experiences of discrimination in any other healthcare setting (not perinatal) | 63.6 | 59.6 | 55.8 | 51.5 | 54.7 | 0.204 |
|  | Ability to provide maternity care to people who have the same racial or ethnic identity as me | 87.0 | 92.2 | 91.4 | 85.1 | 87.9 | 0.044 |
| Personal factors | Deep personal commitment to social and reproductive justice | 83.3 | 85.2 | 84.6 | 83.0 | 83.8 | 0.905 |
| Familial factors | Family members' positive experiences with pregnancy/birth | 4.2 | 18.6 | 19.5 | 17.8 | 17.8 | 0.346 |
|  | Family members' negative experiences with pregnancy/birth | 29.2 | 37.6 | 38.8 | 27.4 | 31.7 | 0.023 |
|  | Family members' experiences of discrimination in a healthcare setting during perinatal period | 16.7 | 37.3 | 38.8 | 27.9 | 31.5 | 0.013 |
|  | Family members' experiences of discrimination in any other healthcare setting (not perinatal) | 25.0 | 37.9 | 39.5 | 30.4 | 33.4 | 0.110 |
|  | Careers of family members | 12.5 | 10.4 | 13.9 | 7.9 | 9.4 | 0.299 |
| Community factors | Community members' positive experiences with pregnancy/birth | 20.8 | 32.2 | 24.7 | 25.7 | 27.4 | 0.241 |
| Community factors | Community members' negative experiences with pregnancy/birth | 45.8 | 51.1 | 41.5 | 45.5 | 46.7 | 0.392 |
|  | Community members' experiences of discrimination in a healthcare setting during perinatal period | 47.8 | 55.7 | 46.9 | 47.0 | 49.6 | 0.171 |
|  | Community members' experiences of discrimination in any other healthcare setting (not perinatal) | 45.8 | 51.7 | 51.2 | 44.4 | 47.3 | 0.281 |
|  | Presence of birth workers and birth justice seekers in my community | 37.5 | 46.2 | 36.6 | 37.9 | 40.2 | 0.168 |
|  | Level of engagement or activism in my community | 29.2 | 39.3 | 30.0 | 29.6 | 32.5 | 0.071 |
| Societal factors | Racial disparities that exist in maternal and child health | 58.3 | 65.7 | 65.9 | 69.0 | 67.4 | 0.603 |
|  | Racial disparities in maternal health care workforce | 54.2 | 63.0 | 63.4 | 68.6 | 66.0 | 0.257 |
| Societal factors | Maternal justice issues | 58.3 | 65.4 | 63.0 | 66.4 | 65.5 | 0.819 |
|  | Reproductive justice issues | 52.2 | 64.4 | 59.8 | 62.8 | 62.7 | 0.640 |
| BARRIERS | | | | | | | |
| Personal barriers | Cost of tuition | 66.7 | 64.4 | 65.0 | 53.2 | 58.2 | 0.014 |
|  | **Cost of books and supplies** | **50.0** | **50.0** | **58.2** | **36.2** | **43.0** | **<0.0001** |
|  | Cost of housing | 41.7 | 39.7 | 36.3 | 31.3 | 34.6 | 0.137 |
|  | Cost of food | 33.3 | 24.7 | 26.3 | 19.7 | 22.3 | 0.181 |
|  | Cost of transportation | 37.5 | 27.4 | 24.4 | 20.1 | 23.3 | 0.055 |
|  | Cost of childcare | 20.8 | 29.1 | 27.2 | 21.4 | 24.2 | 0.138 |
|  | Time commitment | 34.8 | 25.1 | 28.1 | 23.9 | 25.0 | 0.598 |
|  | Travel to and from a school or clinical site | 37.5 | 27.1 | 26.8 | 24.6 | 26.0 | 0.508 |
|  | **My prior educational status** | **25.0** | **23.9** | **16.1** | **8.9** | **14.6** | **<0.0001** |
|  | **My ethnic, cultural, or socioeconomic background** | **45.8** | **20.1** | **30.9** | **14.7** | **18.9** | **<0.0001** |
|  | Reducing work hours or leaving job to study to be a midwife | 50.0 | 34.8 | 42.7 | 41.7 | 40.0 | 0.213 |
|  | Loss of health insurance through job to study to be a midwife | 20.8 | 24.2 | 25.9 | 29.9 | 27.5 | 0.348 |
| Family barriers | Having to find childcare or support with caregiving | 25.0 | 28.1 | 17.1 | 25.8 | 25.5 | 0.271 |
|  | Lack of family support | 8.3 | 13.3 | 11.1 | 10.3 | 11.2 | 0.663 |
| Community barriers | Lack of community knowledge about midwifery | 33.3 | 17.0 | 17.1 | 13.8 | 15.7 | 0.061 |
|  | Lack of community support for midwives and midwifery care | 20.8 | 24.4 | 18.5 | 19.1 | 20.7 | 0.417 |
| Professional community barriers | Lack of current support network to help me pursue midwifery | 20.8 | 23.7 | 28.1 | 17.2 | 20.4 | 0.060 |
|  | Lack of access to other midwifery students or midwives | 33.3 | 23.7 | 29.3 | 18.8 | 21.8 | 0.057 |
|  | Lack of access to mentorship or professional support | 41.7 | 31.7 | 32.9 | 26.1 | 28.9 | 0.159 |
|  | Lack of access to business/entrepreneurial support | 41.7 | 30.9 | 28.1 | 24.0 | 27.1 | 0.091 |
|  | Lack of midwives who have the same racial identity as me | 45.8 | 41.1 | 45.1 | 35.3 | 38.4 | 0.200 |
| Professional barriers | Unsure about job prospects upon graduation | 17.4 | 11.4 | 15.9 | 17.5 | 15.5 | 0.223 |
|  | Unsure about my career path and goals | 8.7 | 7.6 | 11.0 | 10.6 | 9.7 | 0.611 |
|  | Unsure about business or entrepreneurial prospects | 17.4 | 13.1 | 14.6 | 14.6 | 14.3 | 0.915 |
| Educational barriers | **Midwifery school academic prerequisites** | **50.0** | **26.9** | **12.2** | **16.9** | **20.4** | **<0.0001** |
|  | Midwifery school prior experience prerequisites | 20.8 | 16.5 | 17.3 | 10.5 | 13.3 | 0.061 |
|  | Length of program | 25.0 | 14.4 | 17.1 | 16.1 | 16.0 | 0.579 |
|  | Racial inequities in midwifery education/profession | 45.8 | 32.2 | 33.8 | 29.7 | 31.4 | 0.364 |
|  | Lack of scholarships and funding | 58.3 | 57.0 | 56.3 | 49.8 | 52.9 | 0.266 |
|  | Lack of support for English as a second language learners | 4.2 | 2.6 | 3.8 | 2.5 | 2.7 | 0.671^b^ |

^a^ Survey responses were dichotomized as very strong effect or not (i.e., no, slight, moderate, and strong effect).

^b^ *P*-value from Fisher’s exact test.

Note: Bolded entries are significant based on a Bonferroni corrected *p*-value of 0.00031.
